# First vs. multiple cannabis-induced psychotic episodes: Is inpatient treatment any different?

**DOI:** 10.1192/j.eurpsy.2021.1436

**Published:** 2021-08-13

**Authors:** F. Andrade, V. Covelo, A.S. Machado

**Affiliations:** Psychiatric Service, Centro Hospitalar Universitário de São João, Porto, Portugal

**Keywords:** Cannabis, Cannabis psychosis, schizophrénia, FEP

## Abstract

**Introduction:**

Recent studies reported very high cumulative risk for a patient who had cannabis-induced psychosis to be diagnosed with a schizophrenia spectrum disorder.

**Objectives:**

We aim to compare sociodemographic and clinical characteristics, treatment and discharge plan in cannabis-induced first psychosis episode (CI-FEP) vs. multiple cannabis-induced psychotic episodes (CI-MEP) inpatients.

**Methods:**

Retrospective observational study of inpatient episodes with a discharge diagnosis of cannabis-induced psychosis between January 1^st^, 2018 and December 31^st^, 2019 in the Psychiatry Service of CHUSJ. Descriptive analysis of the results was performed using the SPSS software, version 26.0.

**Results:**

Our sample included 61 inpatients, 19 (31.1%) with CI-FEP and 42 (68.9%) with CI-MEP. CI-MEP group had a median of 1±0,234 previous hospital admissions. CI-MEP group has 10,0 higher odds of being discharged in outpatient compulsory treatment (CI 95% 1,21-82,50, p=0,013) and 6.0 odds of being treated with long-acting injectable antipsychotics (LAIAP) (CI 95% 1,79-20,31, p=0,002) when compared to CI-FEP group. Having multiple cannabis-induced psychotic episodes was associated with future admissions to psychiatry unit (OR 4,85 (95% CI 1,23-19,15, p=0,018). We found no statistically significant differences regarding the sociodemographic and clinical characteristics, use habits and discharge plan between the two groups.

**Conclusions:**

Patients with multiple psychotic episodes due to cannabis use are more likely to have a LAIAP prescription, be discharged in compulsory outpatient regimen and be readmitted in to psychiatric inpatient unit. Considering the prevalence of CI-MEP and the risk of chronicity, we need integrative treatment programs to address the specificities of these patients.

